# Enhanced catalytic reduction through in situ synthesized gold nanoparticles embedded in glucosamine/alginate nanocomposites

**DOI:** 10.3762/bjnano.15.99

**Published:** 2024-10-04

**Authors:** Chi-Hien Dang, Le-Kim-Thuy Nguyen, Minh-Trong Tran, Van-Dung Le, Nguyen Minh Ty, T Ngoc Han Pham, Hieu Vu-Quang, Tran Thi Kim Chi, Tran Thi Huong Giang, Nguyen Thi Thanh Tu, Thanh-Danh Nguyen

**Affiliations:** 1 Institute of Chemical Technology, Vietnam Academy of Science and Technology, 1A, TL29, Thanh Loc Ward, District 12, Ho Chi Minh City, Vietnam; 2 Graduate University of Science and Technology, Vietnam Academy of Science and Technology, 18 Hoang Quoc Viet, Cau Giay District, Hanoi, Vietnam; 3 NTT Hi-Tech Institute, Nguyen Tat Thanh University, Ho Chi Minh City 700000, Vietnam; 4 Institute of Materials Science, Vietnam Academy of Science and Technology, 18 Hoang Quoc 14 Viet, Cau Giay District, Hanoi 11000, Vietnam; 5 Faculty of Applied Technology, School of Technology, Van Lang University, Ho Chi Minh City, Vietnam

**Keywords:** catalysis, gold nanoparticles, organic dyes, organometallic nanocomposites, reduction

## Abstract

This study introduces a highly efficient and straightforward method for synthesizing gold nanoparticles (AuNPs) within a glucosamine/alginate (GluN/Alg) nanocomposite via an ionotropic gelation mechanism in aqueous environment. The resulting nanocomposite, AuNPs@GluN/Alg, underwent thorough characterization using UV–vis, EDX, FTIR, SEM, TEM, SAED, and XRD analyses. The spherical AuNPs exhibited uniform size with an average diameter of 10.0 nm. The nanocomposites facilitated the recyclable reduction of organic dyes, including 2-nitrophenol, 4-nitrophenol, and methyl orange, employing NaBH_4_ as the reducing agent. Kinetic studies further underscored the potential of this nanocomposite as a versatile catalyst with promising applications across various industrial sectors.

## Introduction

Gold nanoparticles (AuNPs) have garnered significant attention because of their exceptional physicochemical properties and diverse potential chemical applications [1–3]. The conventional synthesis of AuNPs typically involves the chemical reduction of Au^3+^ ions using various reducing agents and stabilizers [4–5]. However, many of these chemicals are highly reactive, posing risks to both the environment and biological systems. Consequently, integrating green chemistry principles into nanotechnology has become a focus of nanoscience research [6–7]. Numerous studies have highlighted the use of natural compounds or natural sources in the green synthesis of AuNPs [8–9]. Polysaccharides, in particular, have demonstrated significant control over the nucleation and growth of metallic nanoparticles. Utilizing polysaccharide-mediated procedures for AuNP synthesis offers several advantages over conventional methods, including cost-effectiveness, energy efficiency, low toxicity, and eco-friendliness [10–12].

Sodium alginate, derived from marine algae, consists of β-ᴅ-mannuronic acid and its stereoisomer α-ʟ-guluronic acid, forming a linear block copolymer with branched chains [13–14]. This biopolymer possesses metal-binding functional groups that readily cross-link through strong electrostatic bonds with multivalent metal cations (such as Ca^2+^, Ba^2+^, and Cu^2+^) to create an extensive gel network in water [15–16]. The cross-linking of saccharide chains within alginate generates macromolecules ranging in size from micrometers to millimeters, resulting in gelispheres insoluble in water. While gelispheres enhance the physical and mechanical properties of sodium alginate, their poor solubility limits their applicability.

Recently, an ionotropic gelation method has been developed to produce nanoparticles from gelispheres through interaction between oligosaccharides (e.g., cyclodextrins) and the cross-linker [17]. These materials find wide applications in drug delivery [18] and the encapsulation of nanometals [19–20]. Reduction of metallic ions can be achieved using various agents, including plant extracts [21]. This reduction typically involves two steps, namely, the loading of metallic ions onto the nanogel and the subsequent reduction [22–23]. In recent studies, in situ reduction of metal nanoparticles (MNPs) has been explored to enhance synthetic efficiency and streamline procedures by employing disaccharides such as lactose [24–25]. However, the potential of monosaccharides (e.g., glucose, fructose, and glucosamine (GluN)) for the in situ synthesis of metallic nanoparticles onto alginate-based nanogels remains unexplored.

Toxic organic dyes, including nitrophenols and methyl orange (MO), pose a significant environmental threat because of their persistence in water. This challenge has spurred extensive research into various remediation methods. Recently, there has been growing interest in catalytic degradation using oxidizing agents such as H_2_O_2_ and iron in Fenton reactions [26], as well as reducing agents such as NaBH_4_ with nanometal catalysts. Additionally, the reduction of dyes, such as 4-nitrophenol (4-NiP) to harmless 4-aminophenol, a precursor for the synthesis of drugs like paracetamol and phenacetin, shows promise for producing dyes, cosmetics, and pharmaceuticals [27]. Plasmonic MNPs enhance catalytic performance by absorbing both dye molecules and BH_4_^−^ ions, offering a potent solution for dye remediation [28].

In our ongoing research, we synthesized for the first time AuNPs using GluN molecules, serving as both interaction agents to cross-link Ca-Alg gelispheres and reducing agents, thereby providing a novel material for the stabilization of AuNPs. The resulting nanocomposites were characterized using various analytical techniques and demonstrated efficacy in the catalytic reduction of nitrophenols and methyl orange. A comparative analysis of the reduction processes was carried out to confirm the enhanced efficiency of the nanocomposites.

## Experimental

### Materials

The study utilized reagents and chemicals without additional purification. Glucosamine, gold(III) chloride, calcium acetate hydrate, sodium alginate, sodium tetrahydroborate, 2-nitrophenol, 4-nitrophenol, and methyl orange were procured from Acros Scientific (Belgium). Distilled water was used throughout the experimentation.

#### Preparation of nanocomposites

The preparation of the blank GluN/Alg nanocomposite involved an ionotropic gelation as outlined in our recent work [25]. In brief, calcium acetate aqueous solution (7.18 mL; 583 mg·mL^−1^) was gradually mixed with sodium alginate aqueous solution (20 mL; 7.0 mg·mL^−1^) while stirring for 1 h at 1300 rpm. Following overnight settling, the Alg/Ca^2+^ gelispheres underwent centrifugation at 3000 rpm for 10 min and were washed three times with 10 mL of distilled water each time to remove any impurities. Next, an aqueous solution of GluN (12.0 mL; 1.6 mg·mL^−1^) was added to the Alg/Ca^2+^ gelispheres and stirred for 1 h at 1300 rpm. The resulting GluN/Alg suspension underwent ultrasonication for 30 min and was left overnight to ensure the formation of a stable gel. The resulting nanocomposite mixture was centrifuged at 3000 rpm for 10 min and washed three times with 10 mL of water each time to obtain the blank GluN/Alg nanocomposite, which was also used as the standard sample for further characterization. To synthesize the AuNPs@GluN/Alg nanocomposite, aqueous AuCl_3_ solution (13 mL, 0.0061–0.0554 mg·mL^−1^) was added to GluN/Alg suspension (8 mL, 1.0 g·mL^−1^). The reduction process was initiated through heating the mixture and visually confirmed by a change in color of the reaction mixture indicating the formation of AuNPs on the GluN/Alg composite. UV–vis spectroscopy within the range of 300 to 600 nm was employed to monitor this process. Subsequently, the mixture underwent centrifugation at 3000 rpm for 10 min and was washed with distilled water to eliminate impurities and unreacted metal ions. The resulting AuNPs@GluN/Alg nanocomposite powder was dried overnight in an oven at 60 °C and stored for further analysis.

To optimize the reaction parameters, the synthesis of AuNPs@GluN/Alg was fine-tuned to determine the optimal conditions. The impact of the mass ratio between metallic ions and GluN/Alg gel was investigated at levels of 1%, 3%, 5%, 7%, and 9% (w/w). The reaction temperature ranged from 30 to 90 °C, while the reaction time varied from 0 to 140 min.

#### Physicochemical characterizations of AuNPs@GluN/Alg nanocomposite

The samples synthesized under optimal conditions underwent physicochemical characterization and were used for the catalytic reduction of organic dyes. FTIR spectra of blank GluN/Alg and AuNPs@GluN/Alg were acquired using a Bruker Tensor 27 FTIR spectrophotometer, which scanned wavelengths from 500 to 4000 cm^−1^. KBr pellets were used for the measurements. For morphological investigations of the nanocomposites, scanning electron microscopy (SEM) was performed using a SEM-S4800 instrument; transmission electron microscopy (TEM) and selected area electron diffraction (SAED) were carried out using a JEOL JEM-2100 instrument. Crystal structure characterizations of AuNPs were carried out via XRD diffraction. Zeta potential and dynamic light scattering (DLS) measurements were carried out on gel solutions (1.0 mg·mL^−1^) using a nanoPartica Horiba SZ-100 instrument. Thermal analysis through thermogravimetry (TGA) was performed using a LabSys evo S60/58988 Thermoanalyzer (Setaram, France). The oven-dried powder of both blank nanocomposite GluN/Alg and synthesized composite AuNPs@GluN/Alg underwent heating from 30 to 800 °C at a rate of 10 °C·min^−1^ in an airflow of 20 mL·min^−1^ for TGA analysis.

#### Catalytic activity in the reduction of organic dyes

To evaluate the catalytic effectiveness of the synthesized AuNPs@GluN/Alg, we examined their ability to reduce the organic dyes 2-nitrophenol (2-NiP), 4-NiP, and MO using an excess of NaBH_4_. Initially, 3.0 mg of the AuNPs@GluN/Alg solid was placed in a quartz cell with 1 cm path length, along with an aqueous solution of the organic dyes (2.5 mL, 0.1 mM). Then, 0.5 mL of NaBH_4_ solution (0.06 M) was added to start the reaction. The reduction process of the dyes was in situ tracked over time using a UV–vis spectrophotometer, covering the wavelength range of 200–600 nm at room temperature without delay. We examined the kinetics of catalytic degradation by monitoring changes in absorbance values at specific wavelengths.

Given the small quantity of nanocatalyst and the excessive amount of NaBH_4_, we treated the catalytic reduction of these dyes as a pseudo-first-order reaction. The reaction kinetics are described by the equation ln(*C_t_*/*C*_0_) = −*k·t*, where *k* represents the rate constant, and *C*_0_ and *C_t_* denote the initial concentration and the concentration at time *t*, respectively. The rate constant was derived from the slope of the plots of ln(*C_t_*/*C_0_*) as function of the reaction time [29–31].

To assess the reusability of AuNPs@GluN/Alg, we carried out the dye reduction process re-using the nanocomposite. Following each run, the nanocomposite was recovered from the reaction solution, cleaned with ethanol, and then washed multiple times with distilled water before being used again.

## Results and Discussion

### In situ synthesis of AuNPs@Glu/Alg

**Figure 1:**
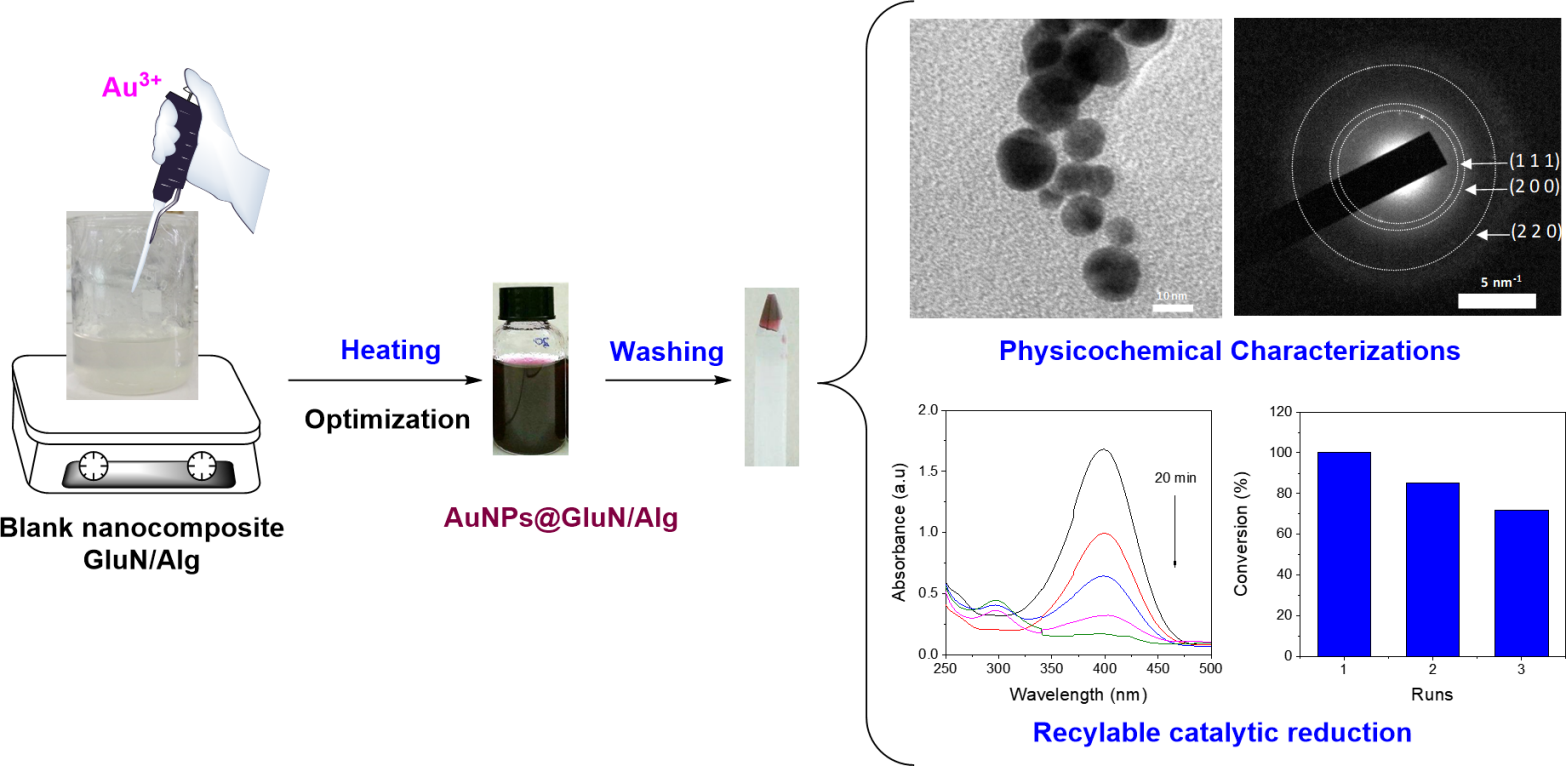
Schematic workflow illustration of the current study.

AuNPs are incorporated into a nanocomposite composed of GluN/Alg following the process depicted in Figure 1. Initially, the blank nanocomposite GluN/Alg is synthesized using the ionotropic gelation method, a technique previously described in our research. GluN serves a dual purpose in this process; it imparts negative charges, disrupting some cross-links within the insoluble gelispheres of Ca–alginate, and acts as a reductant, converting Au^3+^ into Au(0) during the synthesis of the nanocomposite AuNPs@GluN/Alg without additional chemicals. Alginate serves as a stabilizing agent. In the ionotropic gelation mechanism, the cross-linking matrix of Ca–alginate, formed by Ca^2+^ ions and carboxyl groups in sodium alginate, becomes insoluble, while the sugar molecules of GluN interact with Ca^2+^ ions, resulting in the formation of nanostructured particles and forming a homogeneous gel solution of GluN/Alg [24–25]. To synthesize AuNPs@GluN/Alg, the blank nanocomposite is centrifuged to remove impurities before introducing gold ions into the gel solution. The resulting solution is heated to facilitate the in situ reduction of Au^3+^ to Au(0). The synthesis conditions for AuNPs@GluN/Alg were optimized, followed by physicochemical characterizations using various analytical techniques. Finally, the application of the nanocomposite in catalyzing the reduction of organic dyes is explored.

**Figure 2:**
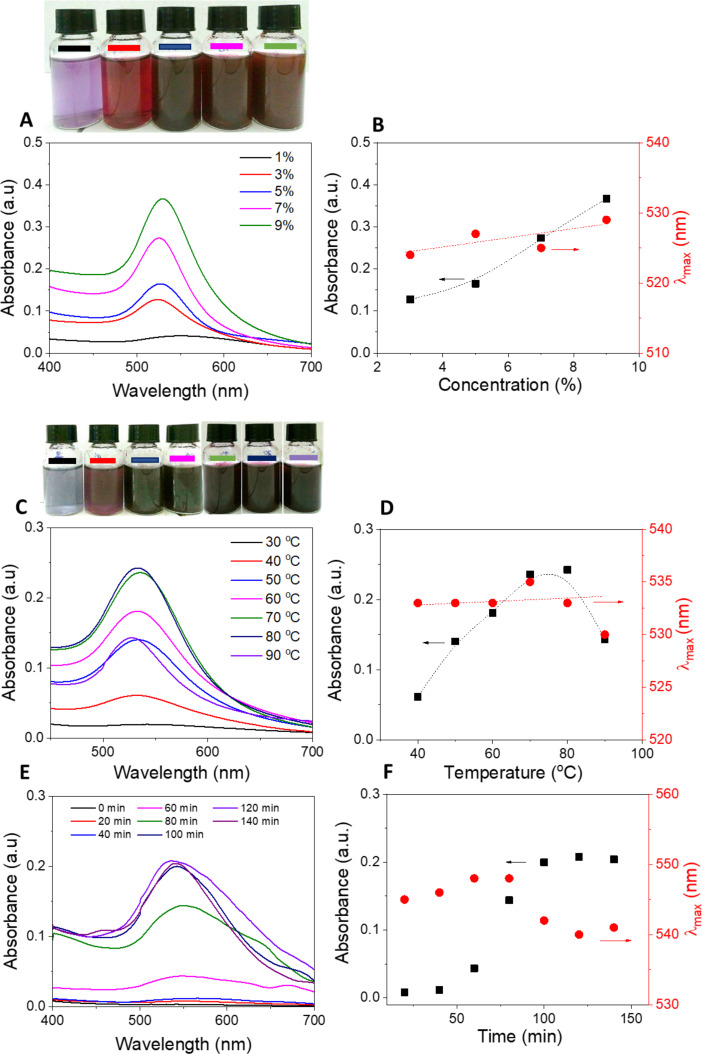
UV–vis spectra of AuNPs@GluN/Alg measured under varying reaction conditions (left) and plots of absorbance as function of various synthesis parameters (right). (A, B) Au^3+^ ions-to-gel ratio (w/w), (C, D) reaction temperature, and (E and F) reaction time. The upper photographs showing the corresponding color change.

Optimizing the synthesis conditions is crucial for the formation of AuNPs@GluN/Alg. In this study, we focused on determining the most favorable parameters for nanocomposite synthesis, including the ratio of Au^3+^ ions to GluN/Alg gel, the reaction temperature, and the reaction time, by using UV–vis spectroscopy. Changes in the physicochemical properties, such as morphology and particle size of AuNPs, were monitored through absorbance and the λ_max_ values of the surface plasmon resonance (SPR) band. Figure 2 illustrates the impact of synthesis conditions on the formation of AuNPs@GluN/Alg. We explored the Au^3+^ ion-to-gel ratios ranging from 1% to 9% (w/w). The results indicate that the concentration of Au^3+^ ions profoundly influences the formation of stable AuNPs. An increase in Au^3+^ concentration led to a corresponding rise in the absorbance values at the SPR band, indicative of increased AuNP formation, while the λ_max_ values exhibited insignificant changes (Figure 2A,B). However, at higher Au^3+^-to-gel ratios (9%), we observed the presence of solid material after synthesis, likely due to nanocomposite aggregation. Therefore, to achieve homogeneous nanoparticles, we opted for an Au^3+^-to-gel ratio of 7% for further investigations.

Figure 2C,D illustrates the significant impact of the reaction temperature on the formation of nanocomposites. As the temperature increases, the UV–vis absorbance rises, reaching a peak at 70 °C, after which the absorbance declines again at higher temperatures (80 and 90 °C). This decrease in AuNP concentration in the colloidal solution is attributed to nanoparticle aggregation. Elevated temperatures can cause the detachment of the polysaccharide chains from the surface of the AuNPs, promoting the collision and attachment of nanoparticles to form larger aggregates, followed by coalescence [32–33]. Additionally, the morphology of AuNPs changes significantly at higher temperatures, as evidenced by a reduction in the λ_max_ values.

The optimization of reaction time was carried out within a range of 0 to 140 min. Figure 2E,F demonstrates the profound effect of the reaction time on AuNP formation under heating at 70 °C. The SPR band of the AuNPs only becomes evident after 40 min of heating, with the absorbance values peaking at 100 min, and the λ_max_ values stabilize after this duration. Thus, the optimal conditions for synthesizing AuNPs@GluN/Alg were determined to be a Au^3+^ ions-to-gel ratio of 7% and heating at 70 °C for 100 min. These optimal samples were further characterized and evaluated regarding their catalytic activity.

### Characterization of AuNPs@GluN/Alg

**Figure 3:**
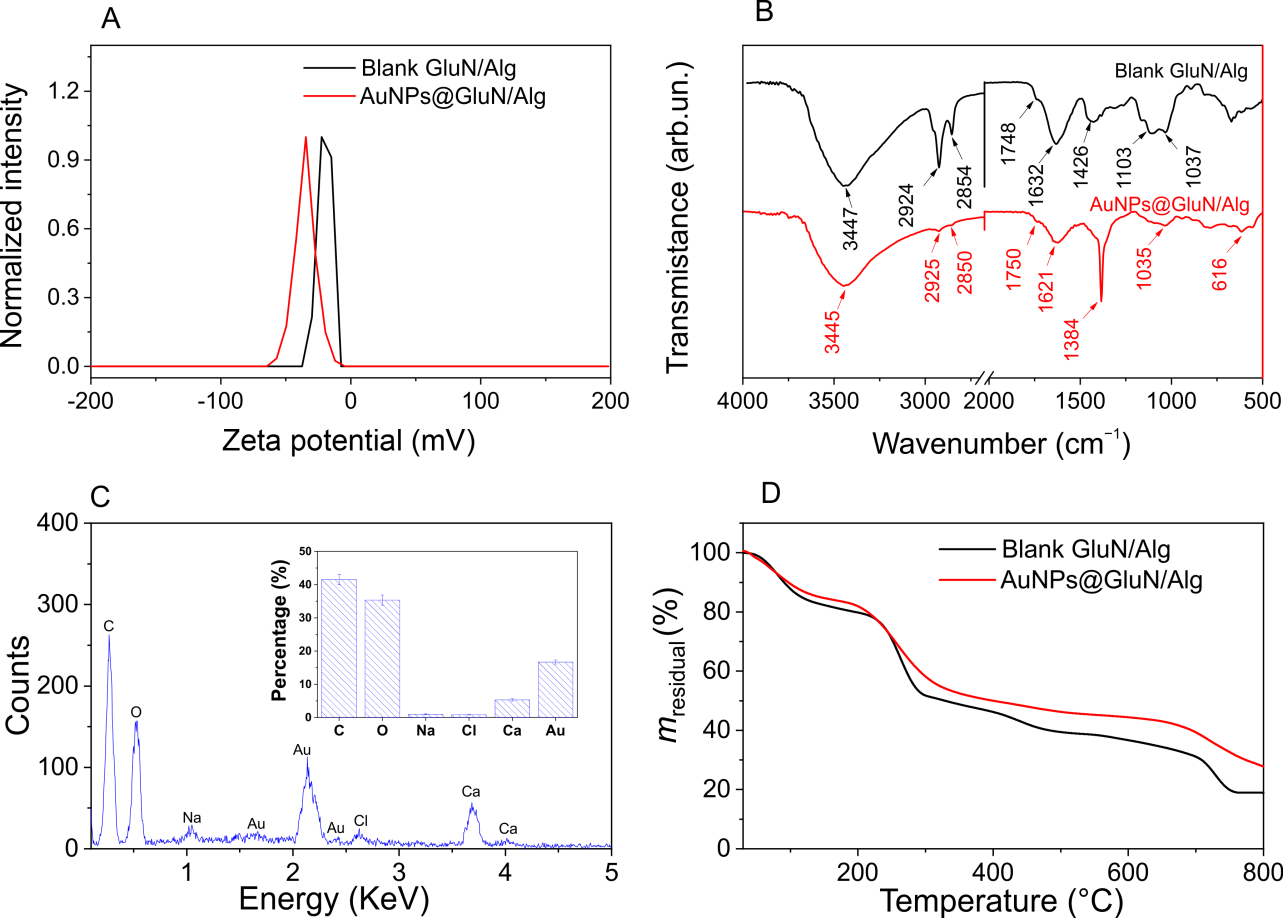
(A) Zeta potential curves, (B) FTIR spectra, (C) EDX spectrum and elemental composition (AuNPs@GluN/Alg only), and (D) TGA curves of GluN/Alg and AuNPs@GluN/Alg nanocomposites.

Zeta potential analysis was employed to assess the stability of nanoparticles in the colloidal solution. Figure 3A shows that both the blank and AuNPs@GluN/Alg nanocomposites exhibit negative zeta potentials of −22 and −35 mV, respectively. The increased negativity in the zeta potential of AuNPs@GluN/Alg is attributed to the presence of AuNPs within the composite. The highly negative zeta potentials of both nanocomposites indicated their high stability in the aqueous solution.

The functional groups present in the nanocomposite were identified through FTIR spectroscopy, with the blank nanocomposite serving as a reference for the analysis of AuNPs@GluN/Alg. Figure 3B illustrates the similarity between the spectra. Peaks in the blank nanocomposite occur at specific wavenumbers (3447, 2924, 2854, 1748, 1632, and 1037 cm^−1^), with slight shifts observed in the corresponding bands of AuNPs@GluN/Alg (3445, 2925, 2850, 1750, 1621, and 1035 cm^−1^). The broad band around 3445 cm^−1^ corresponds to O–H groups of sugar molecules in the polysaccharides, while the vibrations at 2925 and 2850 cm^−1^ are attributed to symmetric stretching of C–H bonds. The peaks at 1750 and 1620 cm^−1^ signify carboxyl groups in alginate chains, with the peak at 1035 cm^−1^ indicating stretching vibrations of C–O bonds [34–35]. The FTIR spectra suggest the critical role of polysaccharides as stabilizing agents for AuNPs within the nanocomposite.

EDX analysis (Figure 3C) was conducted to determine the elemental composition of AuNPs@GluN/Alg. Strong signals at approximately 2.2 and 3.60 keV confirmed the presence of gold and calcium in the sample [36–37]. The identification of calcium reaffirms the formation of cross-links within the nanocomposite. Calculations revealed an average gold fraction of 17.4% (w/w) in the nanocomposite. This high concentration of gold nanoparticles is anticipated to impart excellent catalytic performance in the reduction of organic dyes.

Thermal properties of blank and AuNPs@GluN/Alg nanocomposites were assessed through TGA measurements in an airflow of 20 mL/min and a heating rate of 10 °C/min (Figure 3D). Similar kinds of thermal behavior were observed for both samples, characterized by three stages. At the initial stage, both nanocomposites exhibited a weight loss of approximately 17% in the temperature range of 30–100°C, attributed to the desorption of water absorbed during storage [38]. This showed that the dried nanocomposites can absorb moisture from the ambient environment well. The second stage revealed a slightly higher thermal stability of AuNPs@GluN/Alg (25% mass loss in the range of 210–290 °C) compared to blank GluN/Alg (31% mass loss in the range of 210–320 °C), indicating that AuNPs enhance the thermal stability of the polysaccharides. In the final stage, the weight loss of AuNPs@GluN/Alg was significantly lower than that of the blank nanocomposite in the temperature range of ca. 300–800 °C). Consequently, the ash residual of the AuNPs@GluN/Alg nanocomposite (30% of the initial mass) exceeded that of the blank sample (20% of the initial mass), suggesting the presence of inorganic components within the nanocomposite.

**Figure 4:**
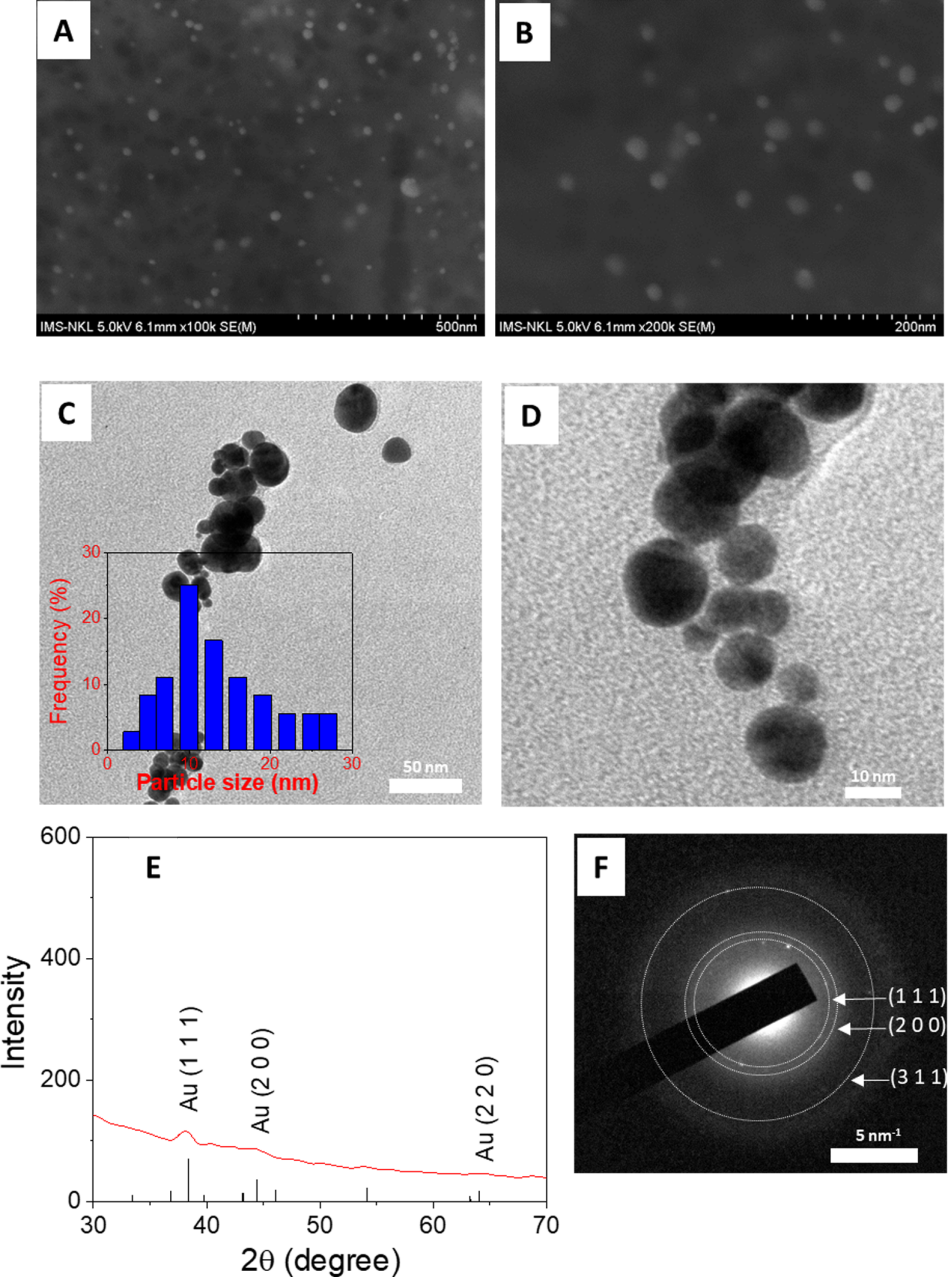
(A, B) SEM images with different magnifications, (C, D) TEM images with different magnifications, and (E) XRD and (F) SAED patterns of the AuNPs@GluN/Alg nanocomposite.

SEM and TEM analyses were performed to determine the morphology, and XRD and SAED patterns were used to evaluate the crystalline structure of AuNPs@GluN/Alg, as illustrated in Figure 4. The SEM images show that the AuNPs are uniform spherical particles with a size below 30 nm (Figure 4A,B). TEM images of AuNPs@GluN/Alg indicate an even geometry of the spherical particles (Figure 4C,D). A narrow size distribution of AuNPs was observed in the range of 3–27 nm with the highest frequency at 10 nm.

The crystalline structure of AuNPs was determined through XRD and SAED analysis. The XRD pattern showed Bragg reflections at 2θ values of 38.1°, 44.1°, and 64.5°, associated to, respectively, the (111), (200), and (220) planes of face-centered cubic (fcc) Au (card no. 96-901-1613) [39–40]. Moreover, the SAED pattern revealed rings of atoms with a d-spacing of 2.38 ± 0.12 Å, 2.92 ± 0.09 Å, and 1.22 ± 0.02 Å, corresponding to, respectively, the (111), (200), and (311) planes of fcc Au. Both analyses confirmed the presence of crystalline AuNPs in the synthesized nanocomposite.

### Catalytic reduction of organic dyes

**Figure 5:**
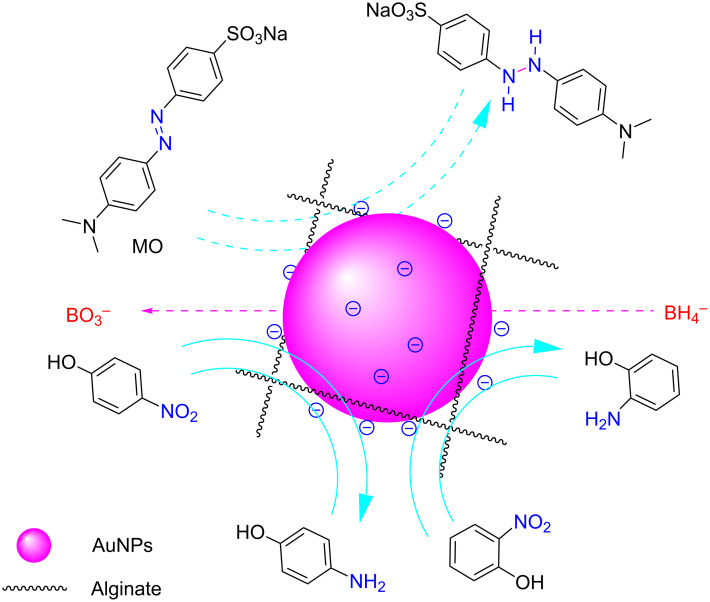
Proposed mechanism of the reduction of 2-NiP, 4-NiP, and MO using NaBH_4_ in the presence of AuNPs@GluN/Alg catalyst.

We investigated the catalytic efficiency of the AuNPs@GluN/Alg nanocomposite in reducing organic dyes using NaBH_4_ as a model reaction. The persistence of toxic organic dyes poses a significant environmental hazard because of their poor biodegradability in aqueous environments [41]. The reduction mechanism involves the transfer of electrons from BH_4_^−^ (the electron donor) to the dye (the electron acceptor) facilitated by the surface of the metal nanoparticles [42–43]. Prior to electron transfer, dye and BH_4_^−^ are adsorbed onto the catalyst surface, as depicted in Figure 5. Consequently, the effectiveness of this reaction hinges on the catalytic surface, influenced by both reagent adsorption and product desorption. We monitored the in situ reduction of organic dyes using the nanocatalyst via time-dependent UV–vis absorption measurements at ambient temperature. Confirmation of reduction was evidenced by the discoloration of the dye solution and the corresponding decrease in absorbance.

**Figure 6:**
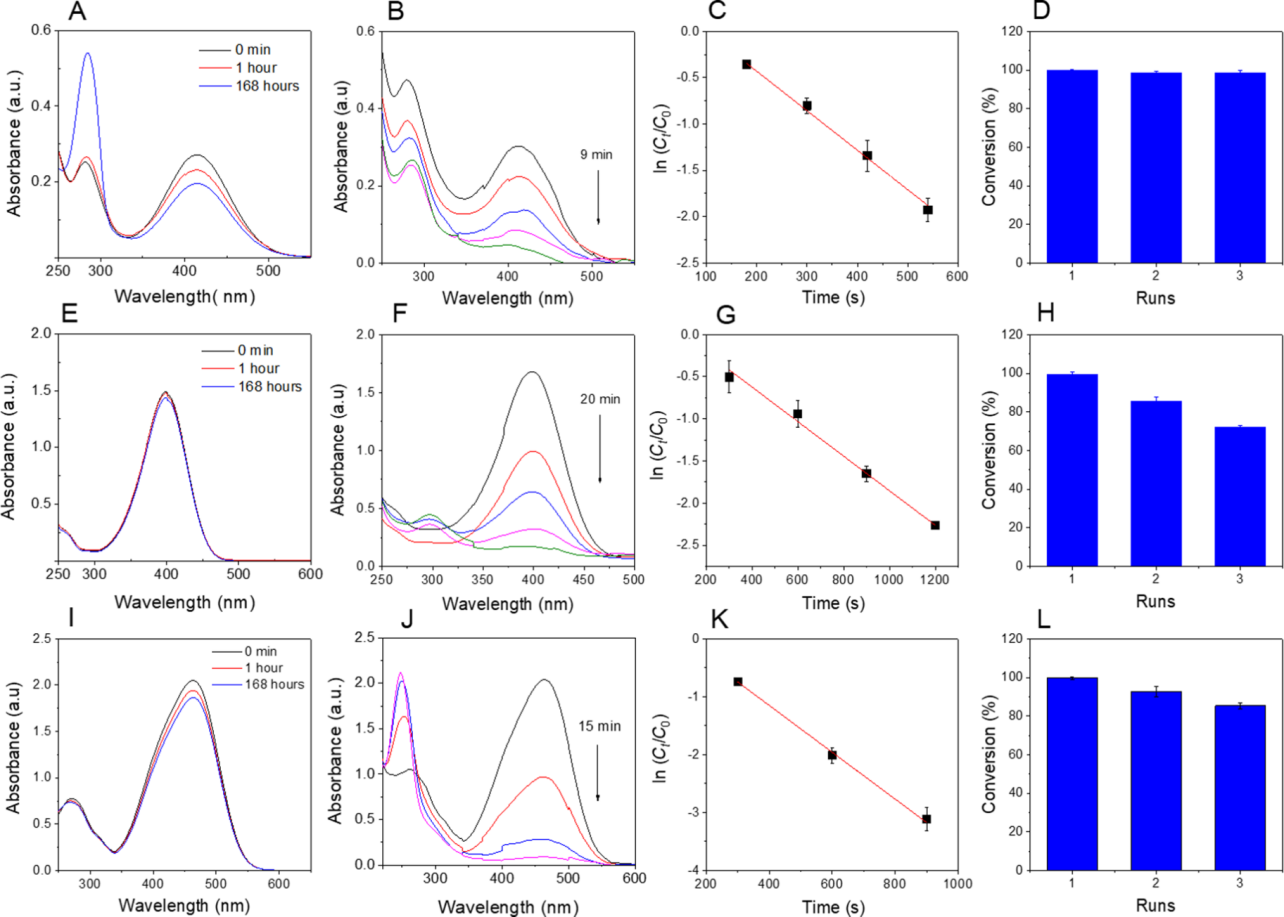
UV–vis spectra during the reduction of 2-NiP (A, B), 4-NiP (E, F), and MO (I, J) without and with nanocatalyst and plots of kinetic data and recyclability regarding 2-NiP (C, D), 4-NiP (G, H), and MO (K, L).

Figure 6 illustrates the results of the reduction of organic dyes. Initial experiments were conducted without the nanocatalyst and monitored over one week (Figure 6A,E,I). The findings revealed a gradual reduction in the concentration of 2-NiP by approximately 15% within the first hour and around 30% over the course of one week. The reduction of the other dyes was minimal in the absence of the nanocatalyst over the same period of time. Intriguingly, when the nanocomposite was introduced, a rapid decrease in absorbance values at specific wavelengths (413 nm for 2-NiP, 400 nm for 4-NiP, and 463 nm for MO) was observed, indicating efficient reduction. The varying reduction rates can be attributed to the structural differences among the dyes. The reduction of 2-NiP reached approximately 65% after the initial 5 min and was nearly complete (ca. 99%) after 9 min. The reduction of 4-NiP and MO took longer, with about 50% reduction after the initial 5 min and reaching near completion (ca. 99%) after 20 and 15 min, respectively. This variance underscores the different rates of dye adsorption and product desorption on the surface of the gold nanoparticles. Additionally, the polysaccharides can interact with functional groups within the dye structure, further influencing the adsorption/desorption dynamics of organic dyes on the surface of the AuNPs [44].

Regarding the reaction kinetics, the plots depicting ln(*C_t_*/*C*_0_) as function of the reaction time exhibit strongly linear correlations, with high determination coefficients, indicating pseudo-first-order kinetics for the degradation of organic dyes (Figure 6C,G,K). The rate constant for the reduction of 2-NiP (4.26 × 10^−3^ s^−1^, *R*^2^ = 0.994) surpassed that for the reduction of 4-NiP (2.05 × 10^−3^ s^−1^, *R*^2^ = 0.995). This variance may stem from the hindrance caused by the interaction between the hydroxy group in 4-NiP and functional groups in the polysaccharide chains, impeding the adsorption/desorption dynamics on the surface of the AuNPs. The rate constant for MO reduction was determined to be 4.04 × 10^−3^ s^−1^, with *R*^2^ = 0.998. Additionally, the recyclability study revealed that the highest recyclability was achieved after the reduction of 2-NiP, with over 98% retention after three cycles, while the recyclability regarding 4-NiP and MO notably declined in the third cycle, with 70% and 80% retention, respectively. These findings underscore the effectiveness of AuNPs@GluN/Alg in facilitating the recyclable reduction of 2-NiP. A comparison of MNPs–alginate-based catalysts shows that the AuNPs@GluN/Alg nanocatalyst significantly enhanced catalytic performance in the reduction of organic dyes (Table 1).

**Table 1:** Comparison of the catalytic activity of alginate-based nanocomposites in the reduction of organic dyes.

Dye	Catalyst	Catalyst mass	Time (min)	Rate constant (s^−1^)	Ref.

2-NiP	TiO_2_–Ag@Alg	10.0 mg	40	0.5 × 10^−3^	[45]
	CeO_2_-SnO@Alg	10.0 mg	9	0.57 × 10^−3^	[46]
	AuNPs@GluN/Alg	3.0 mg	9	4.26 × 10^−3^	this work
4-NiP	AgNPs@PVP/ Alg	—	8	1.23 × 10^−4^	[47]
	AgNPs@HPCD/ Alg	3.0 mg	25	1.51 × 10^−3^	[21]
	PdNPs@HPCD/Alg	5.0 mg	24	1.78 × 10^−3^	[22]
	AuNPs@GluN/Alg	3.0 mg	20	2.05 × 10^−3^	this work
MO	AgNPs@HPCD/ Alg	3.0 mg	22	1.79 × 10^−3^	[21]
	AuNPs@Lac/Alg	3.0 mg	22	2.86 × 10^−3^	[24]
	Cu/Alg-CNBs	3.6 mg	17	2.25 × 10^−3^	[48]
	AuNPs@GluN/Alg	3.0 mg	15	4.04 × 10^−3^	this work

## Conclusion

This study introduces a novel approach for synthesizing gold nanoparticles within glucosamine/alginate nanocomposites via an in situ method. Comprehensive characterization using various analytical techniques was performed, revealing crystalline gold nanoparticles with an average diameter of 10.0 nm. The recyclable catalytic potential of the nanocomposite was then assessed for the reduction of 2-nitrophenol, 4-nitrophenol, and methyl orange utilizing NaBH_4_. Remarkably, the reactions exhibited rapid kinetics, with pseudo-first-order rate constants ranging from 2.05 × 10^−3^ to 4.26 × 10^−3^ s^−1^. Notably, the nanocomposite demonstrated excellent recyclability in the reduction of 2-nitrophenol. Consequently, this innovative nanocomposite emerges as a promising catalyst for organic dye reduction, offering considerable potential for diverse industrial applications.

## Conflicts of Interest

The authors declare that they have no conflicts of interest.

## Data Availability

All data that supports the findings of this study is available in the published article and/or the supporting information to this article.
